# Decoding anaerobes: a comprehensive evaluation of MALDI-TOF Sirius and VITEK MS PRIME in clinical settings

**DOI:** 10.1128/spectrum.01870-25

**Published:** 2025-09-25

**Authors:** Maria Florencia Rocca, Paula Etcheverry, Gastón D´Angiolo, Monica Prieto

**Affiliations:** 1 Servicio Bacteriología Especial, Departamento de Bacteriología, Instituto Nacional de enfermedades infecciosas, INEI-ANLIS "Dr. Carlos G. Malbrán" 62986 , Buenos Aires, Argentina; 2 Red Nacional de identificación microbiológica mediante espectrometría de masas ReNaEM, Buenos Aires, Argentina; University of Maryland School of Medicine, Baltimore, Maryland, USA

**Keywords:** anaerobes, MALDI-TOF, comparative studies, clinical microbiology

## Abstract

The timely identification of anaerobic bacteria at the genus and species levels is critical for managing infections and guiding antimicrobial therapy. Matrix-assisted laser desorption-ionization time-of-flight (MALDI-TOF) mass spectrometry (MS) has emerged as a powerful tool for the identification of anaerobic bacteria, overcoming challenges associated with their special culture requirements and low growth rates. This technique has proven to be both reliable and efficient, providing accurate identification with minimal bacterial biomass. The application of MALDI-TOF MS in clinical settings has significantly improved the identification of anaerobic bacteria, facilitating appropriate treatment decisions and enhancing patient outcomes with minor costs. This study evaluates the performance of two MALDI-TOF mass spectrometry platforms: SIRIUS (Bruker Daltonics) and VITEK MS PRIME (bioMérieux), and their latest libraries, using a panel of 60 clinically relevant anaerobic strains validated in IVD databases and approved by the Food and Drug Administration. Beyond identification accuracy, we highlight the role of rapid confirmation of anaerobic pathogens in improving clinical outcomes. Results are compared against the existing literature, including performance evaluations of our group, to underscore the advancements in MALDI-TOF technology. MALDI-TOF MS has demonstrated high accuracy in identifying anaerobic bacteria, with a genus identification success rate of over 97%, combined with a precision value of 100% and an overall performance agreement of 73.3%, with some minor discrepancies, so the choice of one or the other platform will depend on the needs of each particular laboratory.

**IMPORTANCE:**

Rapid and accurate identification of anaerobic bacteria is essential for guiding antimicrobial therapy and improving patient outcomes, yet it remains challenging due to the organisms’ fastidious nature. Matrix-assisted laser desorption-ionization time-of-flight (MALDI-TOF) mass spectrometry has transformed clinical microbiology by enabling high-throughput, cost-effective, and reliable identification of anaerobes. This study provides a head-to-head comparison of two widely used MALDI-TOF platforms—Bruker SIRIUS and VITEK MS PRIME—using a panel of clinically relevant anaerobic strains. By assessing their diagnostic accuracy, reproducibility, and database performance, our results offer practical insights for laboratories selecting a MALDI-TOF system. The findings have direct implications for improving diagnostic workflows, reducing time-to-result, and enhancing antimicrobial stewardship in clinical settings. Furthermore, this work contributes to the development of national resources and tools that support MALDI-TOF-based diagnostics in low- and middle-income settings.

## INTRODUCTION

Anaerobic bacteria play a significant role in polymicrobial infections, particularly in deep tissue abscesses, bacteremia, and intra-abdominal infections. Rapid identification of these pathogens to the genus and species level is essential for effective management, as anaerobes are often associated with delayed diagnoses due to their challenging growth requirements and identification limitations using conventional methods. Matrix-assisted laser desorption-ionization time-of-flight (MALDI-TOF) mass spectrometry (MS) has revolutionized microbiological diagnostics, offering high-throughput and precise identification capabilities (1). Recent comparative studies of the latest MALDI-TOF MS systems, such as the VITEK MS PRIME (VMSP) and Bruker Biotyper Sirius, demonstrate their robust performance in clinical settings, with both platforms achieving high accuracy for anaerobic and fastidious bacteria (2, 3). These advancements underscore the reliability of MALDI-TOF MS as a frontline diagnostic tool.

In clinical settings, MALDI-TOF MS has been used to identify anaerobic bloodstream infections, with *Bacteroides* spp. and *Clostridium* spp. being the most commonly isolated organisms. The technique has helped identify risk factors for in-hospital mortality, such as age and the presence of solid tumors (4). The identification of anaerobes in clinical samples, such as pus aspirates and tissue samples, has been significant, with common isolates including *Bacteroides fragilis* and *Prevotella* spp. This has implications for evidence-based medicine and antibiotic therapy (5). Specific cases, such as the identification of *Anaerobiospirillum succiniciproducens*, highlight the precision of MALDI-TOF MS. This organism was identified with a score of 2.10, confirmed by 16S rRNA sequencing, demonstrating the method’s reliability in complex cases (6).

While MALDI-TOF MS has proven effective, it is important to note that a small percentage of isolates (2%) may still require molecular methods for final identification (7). This underscores the need for complementary techniques in certain scenarios to ensure comprehensive microbial identification. This study focuses on a comparative evaluation of two MALDI-TOF platforms, the SIRIUS by Bruker Daltonics and the VITEK MS PRIME by bioMérieux, using a panel of 60 isolates of anaerobic strains. The aim is to assess their ability to provide accurate and rapid identification, with a particular emphasis on the clinical relevance of immediate genus- and species-level confirmation, using the latest databases, building upon previous performance studies, including those by our group (8).

## MATERIALS AND METHODS

### Strain selection

Anaerobic species from our reference culture collection included in the k510 Food and Drug Administration validation for IVD databases (https://www.accessdata.fda.gov/scripts/cdrh/cfdocs/cfpmn/pmn.cfm?ID=K163536; https://www.accessdata.fda.gov/scripts/cdrh/cfdocs/cfpmn/pmn.cfm?id=K212461). (9, 10). The sources included deep tissue biopsies, abscesses, and sterile site fluids. All isolates were obtained from monomicrobial cultures.

A total of 60 anaerobic strains were selected, representing genera and species of clinical relevance. These included *Bacteroides* (4)*, Bifidobacterium* (1)*, Clostridium* (9)*, Finegoldia* (2)*, Fusobacterium* (2)*, Lactobacillus* (14)*, Peptoniphilus* (2)*, Peptostreptococcus* (1)*, Porphyromonas* (1)*, Prevotella* (3)*, Propionibacterium* (16), and *Veillonella* (5) as outlined in Table 1.

**TABLE 1:** Clinically relevant microorganisms evaluated in this study and number of isolates tested in duplicate each day, three different days

Anaerobes evaluated	Number of isolates tested
*Bacteroides* spp.	4
*Bifidobacterium* spp.	1
*Clostridium* spp.	9
*Finegoldia magna*	2
*Fusobacterium* spp.	2
*Lactobacillus* spp.	14
*Peptoniphilus asaccharolyticus*	2
*Peptostreptococcus anaerobius*	1
*Porphyromonas asaccharolytica/uenonis*	1
*Prevotella* spp.	3
*Propionibacterium* spp. (*Cutibacterium* spp.)	16
*Veillonella* spp.	5
Total	60

Although *Lactobacillus* species are not considered strict anaerobes, their inclusion in this performance evaluation was justified by their frequent recovery from anaerobic culture conditions and their clinical relevance in polymicrobial infections involving anaerobic flora. In routine diagnostic workflows, particularly when using enriched anaerobic media, these facultative or aerotolerant anaerobes often grow alongside strict anaerobes, necessitating their reliable identification. (11). Therefore, their presence in the testing panel reflects real-world laboratory conditions and contributes to a more comprehensive assessment of MALDI-TOF MS platform performance in identifying clinically relevant anaerobic and microaerophilic bacteria.

### Sample preparation

Strains were cultured under anaerobic conditions following standard protocols: isolates were plated on 5% sheep blood agar plates and incubated for 48 hours under anaerobic conditions at 37°C. The purity of the culture was verified, and the samples were then analyzed using MALDI-TOF MS. According to the national network guidelines, developed after the verification of the identification through several years by reference laboratories in Argentina, all microorganisms were identified using the *in situ* extraction method, which consists of adding 1 µL of formic acid prior to sealing with the commercial hidroxicianocinamicacid (HCCA) matrix. Analyses were performed by duplicate (two spots) on both MALDI-TOF MS instruments: SIRIUS PRIME and VITEK MS PRIME. The assay was conducted by the same operator over 3 consecutive days to assess reproducibility and consistency of results. (12)The entire procedure was developed according to the guide mentioned available at http://sgc.anlis.gob.ar/handle/123456789/2632.

### Instrumentation and databases

On SIRIUS equipment (Bruker Daltonics), the identification was performed using the MBT Compass IVD v.13 database. On VMSP (bioMérieux), the identification was conducted using the Knowledge Base version 3.3.

For identification, the recommendations of each manufacturer were considered as follows: score value ≥2.00 for the species level and ≥1.7 for the genus. Scores values under 1.69 were considered no reliable identification on the SIRIUS platform. When identification was carried out using the VMSP system, values of confidence between 60.0 and 99.9% indicated reliable species identification. Low discrimination occurs when there is more than one significant organism/group, but no more than 4. When there is similarity with more than four organisms or a coincidence is not found, it is considered as No Identification.

To calculate the overall percentage agreement, we considered how many isolates were identified the same (either at the species or genus level) by both platforms. Then, discrepant results, which occurred only with some *Lactobacillus* species/groups, were resolved using 16S rRNA gene sequencing according to CLSI standards for this group. (5)Sequencing and amplification of the 16S rRNA gene were carried out using the primers corresponding to the position 8-27F (5´-AGAGTTTGATYMTGGCTCAG-3´) and 1492R (5´-ACCTTGTTACGACTT-3´) of the 16S rRNA gene of *Escherichia coli,* as described previously (13). PCR products were sequenced using the BigDye Terminator v.3.1 Cycle Sequencing Kit Equipment (Applied Biosystems) and analyzed in the ABI 377 Genetic Analyzer (PE Applied Biosystems). The sequences obtained were compared with standard sequences deposited in the NCBI GenBank (National Center for Biotechnology Information; https://www.ncbi.nlm.nih.gov/genbank/), using the BLAST v.2.0 software (Blast Internet Services, Pittsboro, NC, USA) and interpreted according to CLSI standards.

### Statistical comparison of species-level identification performance

To assess whether the observed difference in species-level identification performance between the two MALDI-TOF MS platforms was statistically significant, McNemar’s test was applied to paired categorical data. A 2 × 2 contingency table was constructed based on the identification outcomes of the 60 tested isolates, comparing the number of correct species-level identifications by each platform.

Then, performance metrics such as identification rates at both the genus and species levels for each platform were evaluated.

## RESULTS

Sixty isolates were evaluated to determine the accuracy, precision, and sensitivity of the VITEK MS PRIME and SIRIUS platforms. The detailed results are described in the supplemental material, then the metrics summary table, including accuracy at the genus and species level where applicable for two MALDI-TOF MS platforms evaluated and their updated databases, is presented in Table 2.

**TABLE 2:** Metrics summary table for two MALDI-TOF MS platforms evaluated and their updated databases

MALDI-TOF MS platform	Accuracy	Precision	Sensitivity	Overall agreement
VMSP	96.7%	100%	96.7%	73.3%
SIRIUS	98.3%	100%	98.3%	

The overall agreement between both platforms was 73.3% (44 isolates). Specifically, agreement at the species level was achieved for 41 isolates, at the genus level for 2 isolates, and 1 isolate remained unidentified by either platform. This results in a genus-level agreement in 43 out of 60 cases (71.7%) and a species-level agreement (68.3%) (Tables S2 and S3).

Both platforms demonstrated very high precision, with no false positives. Bruker SIRIUS showed slightly higher accuracy, correctly identifying one additional isolate at the genus level, but VITEK MS PRIME provided more species-level identifications, which can be clinically valuable in specific cases, such as *Peptoniphilus asaccharolyticus*. While the SIRIUS system identified the microorganism only to the genus level (*Peptoniphilus* spp.), the VITEK MS PRIME system provided a definitive species-level identification. This distinction is crucial, as different species within the genus can exhibit varying virulence and antimicrobial susceptibility profiles. For instance, *P. asaccharolyticus* has been implicated in polymicrobial infections, such as bone and joint infections, and may play a more active pathogenic role than previously recognized (14). Moreover, antimicrobial susceptibility studies have shown that while *P. asaccharolyticus* is generally susceptible to agents like imipenem and metronidazole, resistance to clindamycin and levofloxacin has been observed in certain strains (15).

Therefore, accurate species-level identification, as achieved by VITEK MS PRIME, is essential for guiding effective antimicrobial therapy and improving patient outcomes (16).

In our study, the SIRIUS system successfully identified *Peptostreptococcus anaerobius* at the genus level, whereas the VITEK MS PRIME system failed to identify it in any instance, despite its presence in the database. This discrepancy underscores the variability in performance among MALDI-TOF MS platforms, particularly concerning anaerobic bacteria. This highlights the importance of continuously updating and expanding MALDI-TOF MS databases to enhance the identification accuracy of clinically significant anaerobes (17). Graphical representation of the performance: to complement the statistical analysis, three graphical visualizations were generated to illustrate the performance and concordance between the VITEK MS PRIME and SIRIUS platforms.

Of the 60 clinical isolates evaluated, both platforms correctly identified 41 at the species level. VITEK MS PRIME alone correctly identified 15 isolates that Bruker SIRIUS could not, whereas SIRIUS identified 3 isolates that VITEK failed to classify at the species level. Only one isolate remained unidentified by both systems.

**Fig 1:**
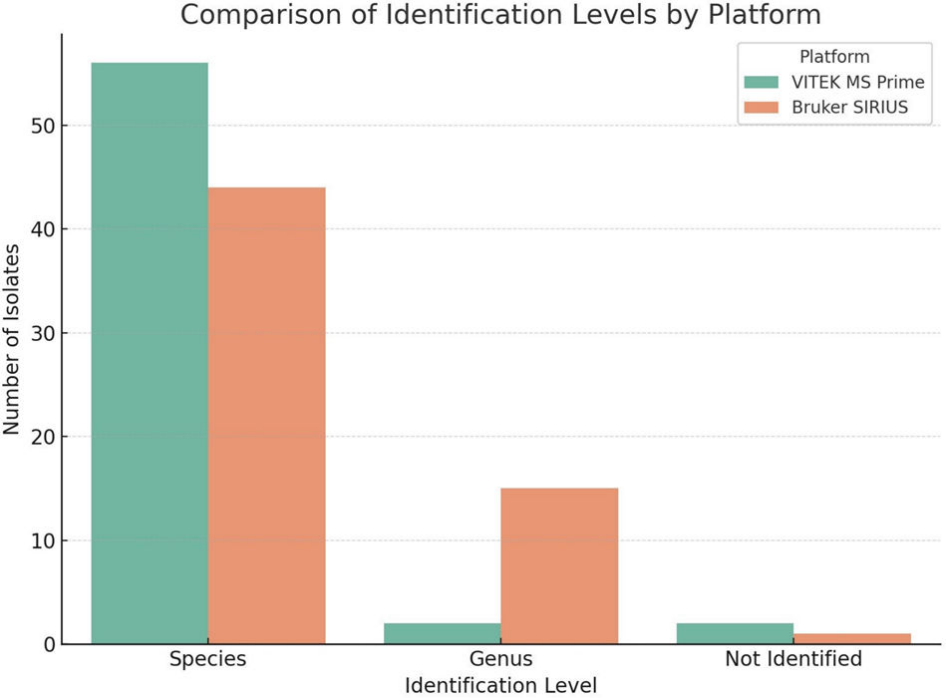
Comparison of the identification levels by platform.

Figure 1 compares the distribution of identifications made by each platform. VITEK MS PRIME identified 56 isolates at the species level, 2 at the genus level, and failed to identify 2 isolates. In contrast, Bruker SIRIUS identified 44 isolates at the species level, 15 at the genus level, and failed to identify 1 isolate.

**Fig 2:**
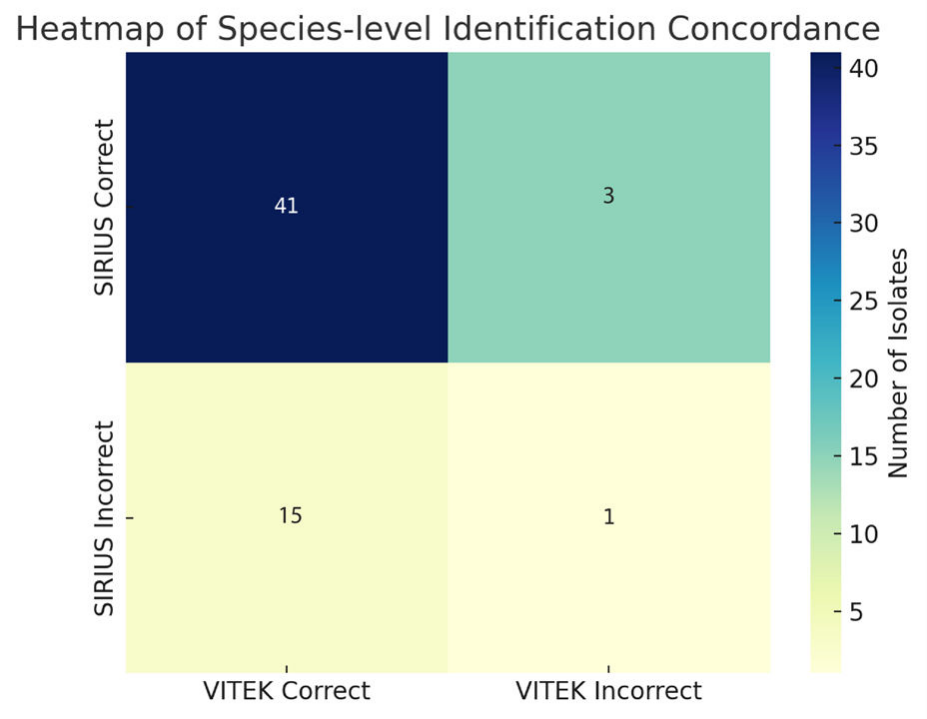
Heat map of species-level identification concordance.

Figure 2 presents a heat map summarizing the species-level identification concordance. The most frequent outcome was agreement between platforms, followed by correct identification by VITEK MS PRIME only. The heat map highlights the asymmetry in performance, which was also confirmed by McNemar’s test.

These graphical summaries reinforce the superior species-level identification capacity of the VITEK MS PRIME system under the tested conditions and emphasize the relevance of integrating visual and statistical tools for performance evaluation (with score values <2.0) and failed to identify only one isolate.

Finally, the overall agreement between platforms was 73.3%, highlighting generally good concordance but also some performance differences, as we describe below.

Discrepancies were primarily observed within the genus *Prevotella*, where both platforms performed excellently with *Prevotella baroniae*. However, the Biotyper system consistently failed to identify *Prevotella veroralis,* with scores under 1.69, which are considered no reliable identification. Within the genus *Veillonella,* VMSP was unable to identify the atypical species, whereas the Biotyper assigned the isolate to the genus level with score values below the 2.0 threshold recommended by the manufacturer. Nevertheless, both platforms correctly identified four isolates of *Veillonella parvula*.

In the case of *Peptoniphilus asaccharolyticus*, the Biotyper system only achieved genus-level identification, while VMSP successfully identified it at the species level. All *Cutibacterium* species evaluated, *C. acnes, C. avidum, and C. namnetense*, were recognized by both systems with high confidence scores, although the Biotyper yielded scores below 2.0 in five instances. Correct species-level identification was also consistently achieved for *Fusobacterium nucleatum, Fusobacterium necrophorum, Finegoldia magna,* and most of the *Lactobacillus* species tested (*L. iners, L. brevis, L. salivarius, L. fermentum, L. jensenii*). However, while VMSP reported clusters such as *Lactobacillus acidophilus/gasseri, Lactobacillus casei/paracasei/rhamnosus*, and *Lactobacillus pentosus/plantarum/paraplantarum,* the Biotyper failed to identify *Lactobacillus buchneri* in any case, despite multiple attempts.

Neither database was able to differentiate between *Bacteroides faecis a*nd *Bacteroides thetaiotaomicron*. However, both platforms correctly identified *B. fragilis* at the species level. In one instance, *Peptostreptococcus anaerobius* was identified at the species level by the Bruker Sirius system, while it was not recognized by the VMSP library. *Porphyromonas asaccharolytica* was identified with high confidence by the IVD library of bioMérieux as *P. asaccharolytica/uenonis*, while the Bruker system produced a score value above 2.0 in only one occasion.

Finally, *Clostridium/Clostridioides* species, including *C. perfringens, C. septicum, C. tertium, C. fallax, and C. difficile,* were all accurately identified. Regarding isolates not identified by either system, these accounted for an average of 6 out of every 60 tested. However, all were correctly resolved upon repeat testing. This outcome is attributed to the reproducibility evaluation protocol employed in our study, in which each isolate was tested on 3 consecutive days. As a result, isolates that initially failed identification due to poor-quality spectra or low confidence scores were successfully identified upon retesting. This approach highlights the importance of repeated analysis to overcome transient technical limitations and ensure reliable species-level identification in routine clinical workflows.

To improve future performance, it is essential to evaluate and optimize technical conditions in the SIRIUS system to reduce the occurrence of “non-peaks,” which directly impacts identification outcomes. Additionally, further work is needed to analyze and fine-tune the confidence thresholds (percentages and score values) in both platforms to enhance identification reliability. While VMSP IVD demonstrated greater reproducibility, it may still introduce errors in fine-grained taxonomic resolution.(18) In contrast, SIRIUS shows greater dependence on spectral peak quality, which, although potentially more accurate in some cases, affects its consistency when spectra quality is suboptimal.

### Statistical comparison of species-level identification performance

Among the isolates tested, both systems correctly identified 41 isolates at the species level. VITEK MS PRIME correctly identified 15 isolates that Bruker SIRIUS failed to classify at the species level, while SIRIUS succeeded in identifying three isolates that VITEK MS PRIME did not. One isolate was not correctly identified by either platform. The resulting McNemar’s test yielded a chi-square value of 8.0 (*P* ≈ 0.0047), indicating a statistically significant difference in performance. These results support the conclusion that VITEK MS PRIME had a higher accuracy in species-level identification compared to Bruker SIRIUS under the conditions of this evaluation.

## DISCUSSION

The findings of this study highlight both the strengths and limitations of current MALDI-TOF platforms in the identification of anaerobic pathogens (19). Our results align with recent evaluations comparing VITEK MS PRIME and Biotyper Sirius, which reported similar discrepancies in species-level identification rates for anaerobes, particularly for genera like *Prevotella* and *Peptoniphilus* (3, 20). While VITEK MS PRIME excelled in reproducibility, observed in the lower number of poor-quality spectra reducing the need for repeat analyses, the Biotyper Sirius occasionally outperformed in taxonomic resolution for select taxa, emphasizing the need for platform-specific optimization (2).

While SIRIUS and VITEK MS PRIME are capable of providing genus- and species-level identifications, their performance varied significantly across the isolates tested. Notably, VITEK MS PRIME demonstrated higher reproducibility and consistency in repeated identifications, even though it failed to identify certain species, such as *Peptostreptococcus anaerobius*. Conversely, SIRIUS showed better coverage for select taxa but was more affected by spectral quality, resulting in occasional failure to generate peaks or low-confidence matches. Both systems showed a moderate-to-high level of concordance, but SIRIUS exhibited lower specificity and was more sensitive to technical variables such as spectrum quality. These discrepancies underline the importance of optimizing technical protocols and database parameters and suggest that MALDI-TOF identification should be complemented with reference methods (e.g., 16S rRNA sequencing) in critical or ambiguous cases. Ultimately, system selection should be guided by the clinical context and laboratory needs, balancing throughput, reproducibility, and taxonomic resolution. Rapid identification of anaerobic pathogens facilitates prompt initiation of targeted antimicrobial therapy, reducing patient morbidity and healthcare costs. The clinical utility of MALDI-TOF is further reinforced by its alignment with molecular gold-standard methods, such as 16S rRNA sequencing. Future studies should explore its application in direct-from-sample workflows to further reduce diagnostic turnaround times.

### Economic impact

The use of MALDI-TOF for the identification of anaerobic pathogens offers significant cost savings for clinical laboratories. Traditional methods, such as biochemical testing or molecular approaches, often require additional reagents, labor-intensive procedures, and extended turnaround times, leading to higher operational costs. MALDI-TOF systems reduce these expenses by enabling rapid, high-throughput identification with minimal consumable requirements. In our experience—albeit anecdotal and not derived from a formal cost analysis—consistent with previous studies (21, 22), MALDI-TOF can reduce per-sample costs by up to 60% and shorten identification times by 24–72 hours compared to biochemical methods. However, it is important to acknowledge the substantial upfront investment required for the instrumentation. The acquisition cost of a MALDI-TOF MS system typically ranges from USD 150,000 to 300,000, depending on the manufacturer, configuration, and service agreements. This high initial expense can be a limiting factor, particularly for small laboratories or institutions in low- and middle-income countries. When amortized over time and weighed against the reduced cost of consumables, labor, and time compared to conventional biochemical or molecular methods, the long-term economic benefits remain significant. Moreover, the scalability and high throughput of MALDI-TOF MS make it particularly cost-effective in laboratories processing large volumes of microbial identifications.

In line with previous studies, the implementation of MALDI-TOF MS for the rapid identification of anaerobic bacteria has been associated with improvements in clinical decision-making and, in some contexts, better patient outcomes (4, 16). While our study did not directly assess clinical endpoints, the capacity of MALDI-TOF to provide timely and accurate species-level identification supports early optimization of antimicrobial therapy and the reduction of unnecessary broad-spectrum antibiotic use, which are key factors influencing patient care. By providing accurate and timely species-level identification, this technique allows for the early optimization of antimicrobial therapy, reducing the empirical use of broad-spectrum antibiotics and minimizing the risk of resistance development.

### Conclusion

This study underscores the importance of MALDI-TOF mass spectrometry in the rapid and accurate identification of anaerobic pathogens. Both SIRIUS and VITEK MS PRIME platforms are valuable tools for clinical microbiology, with complementary strengths that enhance the diagnostic landscape. These findings align with and expand upon previous evaluations, including those by Rocca et al., illustrating the ongoing evolution of MALDI-TOF technology in clinical diagnostics. The results of this experience are being transferred to participants of the Argentinian MALDI-TOF network (RENAEM, Red Nacional de Identificación microbiológica por Espectrometría de Masas MALDI-TOF, http://www.anlis.gov.ar/renaem/).

These findings are also being incorporated into the virtual assistant MALDI BOT, developed by Prieto, Rocca, and Palotay, which is currently under user validation testing. In addition, the information contributes to the open-access guide for the interpretation of MALDI-TOF results, freely available to clinical laboratories at the following link: https://sgc.anlis.gob.ar/handle/123456789/2627 (23).

These tools aim to strengthen interpretation, decision-making, and collaborative knowledge-sharing within the national MALDI-TOF diagnostic network in Argentina.

## SUPPLEMENTAL MATERIAL

Table S1 spectrum.01870-25-s0001.xlsx Identification results of all the anaerobes microorganisms (N:60) tested three days each and in two MALDITOF MS platforms: VITEK MS PRIME and SIRIUS.

Table S2 spectrum.01870-25-s0002.xlsx The 41 isolates for which both platforms provided concordant species-level identification, including organism name, score values (SIRIUS), and confidence percentages (VITEK MS PRIME).

Table S3 spectrum.01870-25-s0003.xlsx The 19 isolates for which species-level agreement was not achieved, including the identification and score/confidence values reported by each platform across all three replicates.

ASM does not own the copyrights to Supplemental Material that may be linked to, or accessed through, an article. The authors have granted ASM a non-exclusive, world-wide license to publish the Supplemental Material files. Please contact the corresponding author directly for reuse.
